# X-Rays in Cancer

**Published:** 1902-12-13

**Authors:** 


### X-RAYS IN CANCER.

At the last meeting of the Pathological Society two papers were read on the X-ray treatment of carcinoma. The first was by Mr. S. G. Shattock, who had carefully examined a scirrhus cancer of the breast which had been removed after being treated by the X-rays, the length of exposure having been gradually increased up to three-quarters of an hour. After 50 exposures the tumour was judged to be somewhat reduced, and a week later the breast was removed. At no time had the skin shown any reaction. After giving the results of a very careful examination of the tumour Mr. Shattock expressed his own feeling of profound disappointment at such a negative histological finding as he had to report; for no degeneration of any kind or cell lysis had been induced in the carcinomatous epithelium, no phagocytic invasion of the epithelium was in progress, and on every side the growth was in an extending condition. There was nothing in the amount of lymphocytic infiltration, in the mast cells, and Unna plasma cells, which might not be met with in cases where no treatment had been carried out. The diminution in size of the mass and the loosening of its deep connections which had been noticed clinically, were difficult to explain, but possibly they were due to the disappearance of a sub-inflammatory œdema. Disappointing as these results were in regard to the hoped-for action of the X-rays upon tissues deeply placed and screened by the skin or mucous membranes, the results of treatment in X-ray treatment in rodent ulcer and cutaneous forms of carcinoma were, Mr. Shattock said, thoroughly established. Of this fact, the second paper, which was read by Mr. Mayou, was a good illustration. He gave a demonstration of the histological changes produced in rodent ulcer by X-ray treatment, changes which apparently led to a cure. A distinct process of inflammation was set up, leucocytic infiltration occurred to a marked degree around the clumps of epithelium, the cells of which became vacuolated and ultimately necrosed. The inflammation, which it was noted was greatly increased by simultaneously dressing the ulcer with irritants such as a 1 to 20 carbolic lotion, did not reach its height until one to three weeks after the commencement of the treatment. A comparison of these two papers strongly suggests that success has something to do with the occurrence of "reaction."

